# Deciphering the Influence of Ground-State Distributions on the Calculation of Photolysis Observables

**DOI:** 10.1021/acs.jpca.3c02333

**Published:** 2023-08-09

**Authors:** Antonio Prlj, Daniel Hollas, Basile F. E. Curchod

**Affiliations:** † Centre for Computational Chemistry, School of Chemistry, University of Bristol, Bristol BS8 1TS, U.K.; ‡ Division of Physical Chemistry, Ruđer Bošković Institute, Zagreb 10000, Croatia

## Abstract

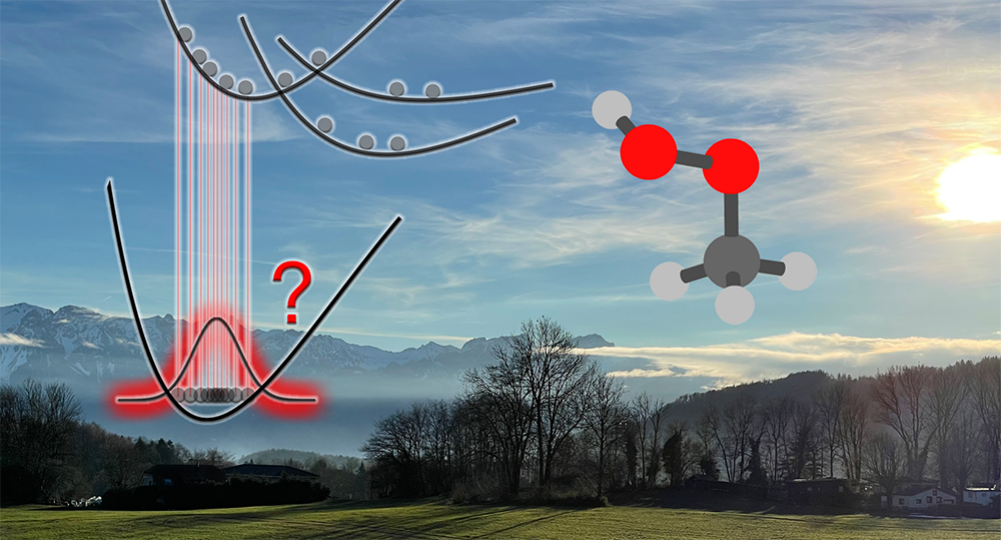

Nonadiabatic molecular dynamics offers a powerful tool for studying the photochemistry of molecular systems. Key to any nonadiabatic molecular dynamics simulation is the definition of its *initial conditions* (ICs), ideally representing the initial molecular quantum state of the system of interest. In this work, we provide a detailed analysis of how ICs may influence the calculation of experimental observables by focusing on the photochemistry of methylhydroperoxide (MHP), the simplest and most abundant organic peroxide in our atmosphere. We investigate the outcome of trajectory surface hopping simulations for distinct sets of ICs sampled from different approximate quantum distributions, namely harmonic Wigner functions and ab initio molecular dynamics using a quantum thermostat (QT). Calculating photoabsorption cross-sections, quantum yields, and translational kinetic energy maps from the results of these simulations reveals the significant effect of the ICs, in particular when low-frequency (∼ a few hundred cm^–1^) normal modes are connected to the photophysics of the molecule. Overall, our results indicate that sampling ICs from ab initio molecular dynamics using a QT is preferable for flexible molecules with photoactive low-frequency modes. From a photochemical perspective, our nonadiabatic dynamics simulations offer an explanation for a low-energy tail observed at high excitation energy in the translational kinetic energy map of MHP.

## 1 Introduction

Nonadiabatic molecular dynamics has become a widely used tool to explore molecular photochemistry and predict relevant photochemical observables. Its applications range, for example, from time-resolved photoelectron spectroscopy,^1,2^ electron diffraction,^3,4^ or ultrafast X-ray scattering^5,6^ to time-independent observables such as translational energy distributions^7^ and quantum yields of photochemical reactions.^8,9^ The in silico prediction of experimental observables is particularly useful for molecular systems where experimental measurements are difficult to interpret or sometimes even challenging to conduct. A typical example of the latter is given by transient volatile organic compounds (VOCs). These molecules are of key importance for atmospheric chemistry,^10−12^ but they are notoriously difficult to study experimentally due to their reactivity and short lifetime. As some VOCs can interact with light and undergo photolysis, ab initio photochemical tools can be readily employed to estimate observables that are important in atmospheric modeling.^13^ In particular, the rate coefficient *J* for a first-order photolytic process can be evaluated as *J* = ∫_λ_min__^λ_max_^ σ(λ) ϕ(λ) *F*(λ) *d*λ, where σ(λ) is the photoabsorption cross-section of the molecule, ϕ(λ) is the photolysis wavelength-dependent quantum yield, *F*(λ) is the photon flux of the light source (actinic flux when the source is sunlight), and λ the wavelength. Both σ(λ) and ϕ(λ) are time-independent observables that can be predicted with state-of-the-art computational methods. Our group has recently proposed a protocol^13^ to determine σ(λ) using the nuclear ensemble approach (NEA)^14^ and ϕ(λ) by resorting to trajectory surface hopping (TSH) dynamics.^15^ This protocol has been applied to VOCs like *tert*-butyl hydroperoxide,^13^ 2-hydroperoxypropanal,^16^ or pyruvic acid.^17^ TSH simulations and the NEA were also used to successfully unravel the photochemistry of other atmospheric molecules (for examples, see refs (18−24)).

The NEA and TSH are among the most popular computational methods used to study the photophysics and photochemistry of medium-size molecular systems.^25^ Both strategies rely on the determination of a ground-state nuclear density to sample a set of discrete phase-space initial conditions (ICs), i.e., nuclear coordinates and momenta. Using the geometries of hundreds of ICs, the NEA proposes to calculate their electronic transitions and broaden them with appropriate shape functions to obtain a convoluted photoabsorption cross-section – σ(λ) – that accounts for non–Condon effects. TSH trajectories are commonly initiated from the same pool of ICs used for the NEA. TSH is a mixed quantum-classical approach in which a swarm of classical trajectories evolves in multiple electronic states with the possibility of inter-state hopping.^15^ It can be employed for systems that are excited instantaneously by an ultrashort laser pulse as well as by continuum-wave fields such as solar irradiation.^26^ As trajectories may have various fates and yield various photoproducts, the different resulting quantum yields, ϕ, are typically evaluated by counting the trajectories giving a certain product and dividing their total number (*N*_product_) by the total number of trajectories in a swarm (*N*_tot_). If wavelength-dependent quantum yields, ϕ(λ), are needed, the ICs for TSH dynamics can be selected from narrow excitation-energy windows in σ(λ), centered around different λ values.^13^

What are then the strategies available to map a ground-state nuclear probability density distribution into ICs? Earlier works commonly used Boltzmann (thermal) sampling by running a long Born–Oppenheimer ground-state dynamics and taking a large number of snapshots as ICs for the NEA (geometries) and the TSH dynamics (geometries and momenta). However, as stressed by Barbatti and Sen,^27^ Boltzmann sampling does not fully recover the zero-point energy (ZPE), resulting in NEA absorption bands that are typically too narrow when compared to experimental data. The outcome of TSH dynamics can also be affected by Boltzmann sampling, leading, for instance, to an extension of the timescales of nonradiative decay and a change in the distribution of reaction pathways.^27^ A Wigner distribution is a more rigorous way to map quantum nuclear densities on quasi phase-space quantities, recovering the quantum delocalization of nuclei and zero-point vibrational effects naturally. Sampling ICs from a Wigner distribution is a common strategy used in many recent works related to excited-state dynamics, but its drawbacks have also been scrutinized.^28−31^ When dealing with realistic multidimensional molecular systems, the Wigner distribution is commonly implemented within the harmonic approximation for uncoupled normal modes, which restricts its reliability to molecular systems with limited anharmonicity. Furthermore, linear normal modes poorly represent torsional degrees of freedom, typically resulting in light atoms being artificially displaced.^28,29,31^ Viable ad hoc corrections resort to filtering out the “problematic” low-frequency modes from the Wigner distribution.^32,33^

As an alternative to Wigner sampling, Suchan et al.^30^ advocated the use of a quantum thermostat (QT) in ground-state dynamics to sample ICs. QT^34−36^ is based on a generalized Langevin equation (GLE) thermostat that keeps the normal modes of a molecular system at different frequency-dependent temperatures—as such, QT provides phase-space distributions corresponding to quantum harmonic oscillators. QT can properly treat both high and low-frequency modes and performs well even for (moderately) anharmonic systems.^30,37^ We note that a different implementation of a similar idea was proposed by Dammak et al.^38^ and termed quantum thermal bath (QTB). QTB was applied to studying vibrational spectra^39^ or the structure of liquid water.^40,41^ A recent extension of QTB was devised to tackle the ZPE leakage issue.^42,43^ Our group has recently compared the impact of QT and Wigner sampling on the prediction of photoabsorption cross-sections of several exemplary VOCs within the NEA.^44^ QT was found superior whenever low-frequency anharmonic modes play a role in the photochemistry/photophysics of a molecule.

One can ask a reasonable question at this stage: what is the influence of the different strategies to sample ICs on the observables predicted by ab initio simulations? In this work, we propose to investigate the impact that Wigner and QT sampling strategies may have on the prediction of a series of experimental observables for methylhydroperoxide (MHP) – CH_3_OOH. MHP is a VOC relevant to atmospheric chemistry^45^ that, despite its simple structure, poses numerous challenges to computational photochemistry. The observables of interest in this work are photoabsorption cross-sections (σ(λ)), wavelength-dependent quantum yields (ϕ(λ)), and translational kinetic energy distributions, predicted from the NEA and TSH simulations based on XMS-CASPT2 electronic structure (see Section 2).

## 2 Computational Details

### 2.1 Electronic-Structure Methods

**Figure 1:**
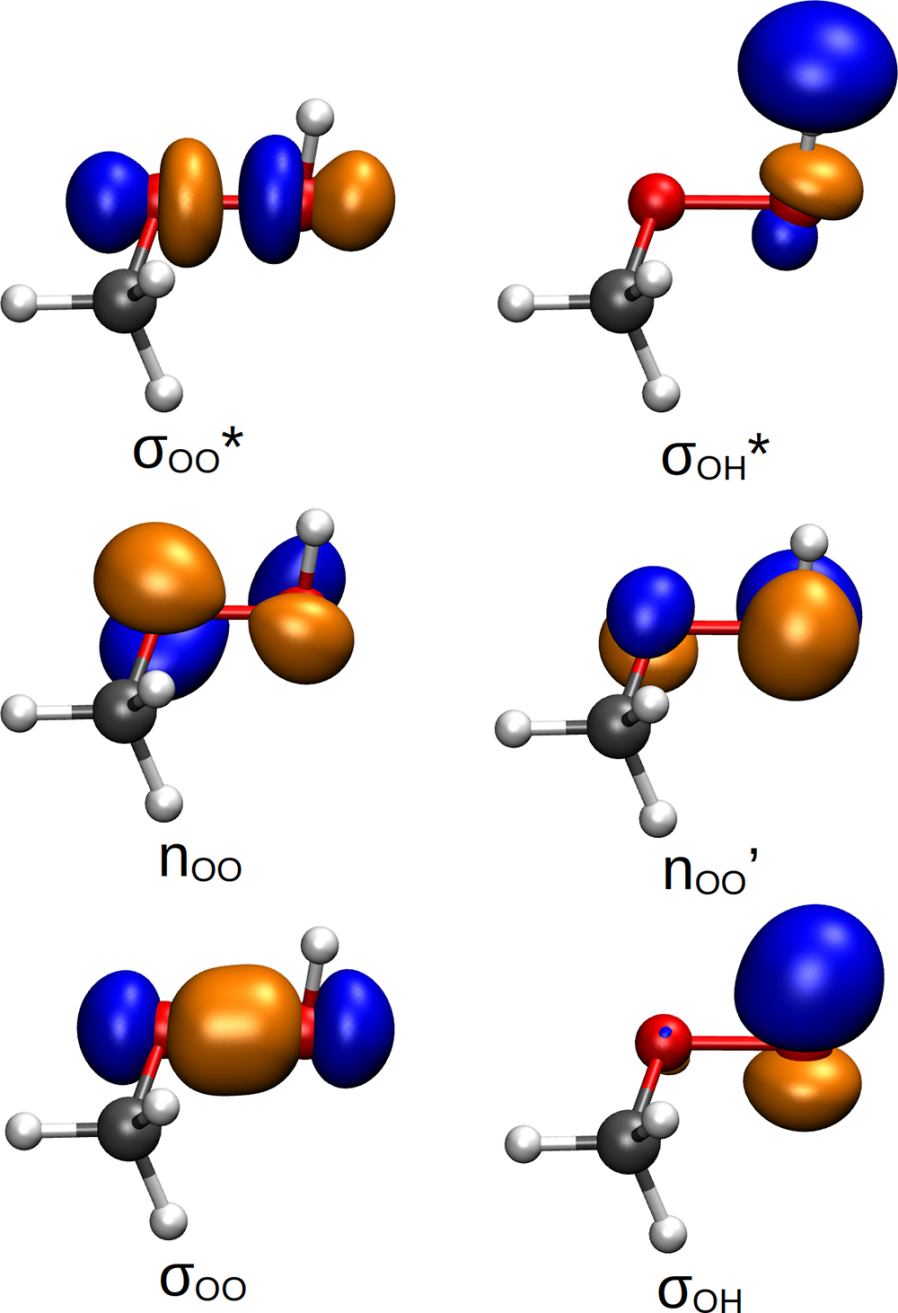
Active space orbitals employed in the XMS(4)-CASPT2(8/6)/def2-SVPD calculations, given here for the ground-state optimized geometry obtained with MP2/aug-cc-pVDZ. Isovalue was set to 0.1.

The ground and three lowest excited electronic singlet states of MHP were calculated with extended multi-state complete active space second-order perturbation theory (XMS-CASPT2)^46^ using BAGEL 1.2.0 package.^47^ The choice of the multireference method XMS-CASPT2 is dictated by the fact that the photodissociation dynamics (i.e., bond breaking) of a molecule bearing a hydroperoxy group cannot be properly described by a single-reference method such as LR-TDDFT or ADC(2).^13^ We employed XMS(4)-CASPT2(8/6) along with a def2-SVPD basis set,^48^ where the active space was composed of six orbitals, two nonbonding *n* orbitals localized on O atoms and two pairs of bonding and antibonding σ/σ* orbitals describing O–O and O–H bonds (see Figure 1). In contrast to earlier work on *tert*-butylhydroperoxide^13^ (see Figure S3 in ref (13)), we excluded the σ/σ* orbitals of the C–O bond as they proved to have no impact on low-lying electronic states and ensuing nonadiabatic molecular dynamics (for both *tert*-butylhydroperoxide and MHP). XMS-CASPT2 was employed within the single state–single reference (SS-SR) contraction scheme.^49^ A real vertical shift was set to 0.5 a.u. to avoid problems with intruder states. Similar values for the vertical shift were used in earlier XMS-CASPT2-based TSH simulations.^13,50^ We found that this shift value increases the numerical stability of the TSH dynamics of MHP even though it slightly deteriorates excitation energies and oscillator strengths (see Table S3). Frozen core and density-fitting approximations (using the def2-TZVPP-jkfit basis set from the BAGEL library) were applied. A detailed benchmark of the electronic energies and oscillator strengths with other electronic structure methods, including the high-level CC3 reference,^51,52^ is given in the SI. Orbitals and molecular representations were visualized with the VMD package, version 1.9.3.^53^

### 2.2 Ground-State Sampling and Photoabsorption Cross-Sections

A harmonic Wigner distribution was calculated with the SHARC 2.1 package^54,55^ using all the harmonic normal mode frequencies obtained for the ground-state minimum-energy structure. To investigate the effect of low-frequency modes, it is possible to omit certain vibrational modes from the harmonic Wigner sampling. In the specific case of MHP, we performed a Wigner sampling without the lowest-frequency normal mode, corresponding to the C–O–O–H torsion. We refer to this distribution as Wigner*. Geometry optimizations and normal-mode calculations were performed with Turbomole 7.4.1.^56^ at the MP2/aug-cc-pVDZ level of theory (see MP2 benchmark in ref (57) as well as SI of ref (13)). MP2/aug-cc-pVDZ provides geometries that are very similar to those obtained with XMS-CASPT2/def2-SVPD,^13^ while it can be used for a long ground-state dynamics simulations needed for QT sampling.

QT sampling was performed with the ABIN code,^58^ coupled to Turbomole for the electronic structure. The GLE thermostat parameters in the form of drift matrix **A** and diffusion matrix **C** were taken from the GLE4MD web page^59^ using a target temperature *T* = 298 K, number of additional degrees of freedom *N*_s_ = 6, *ℏ*ω_max_/*kT* = 20, and the strong coupling regime to prevent issues with ZPE leakage.^35^ ω_max_ corresponds to the maximum normal mode frequency for which the GLE parameters were optimized for a given temperature *T*. For *T* = 298 K, the maximum frequency evaluates to 4114 cm^–1^, which is well above the largest frequency in MHP (3756 cm^–1^ at the MP2/aug-cc-pVDZ level of theory). A time step of ∼0.5 fs was used in molecular dynamics, and the equilibration time was determined by monitoring the convergence of the average kinetic energy temperature. To benchmark the QT distributions in the coordinate space, we also performed path integral simulations combined with the GLE thermostat within the PI+GLE method.^60^ This strategy converges to the exact quantum results faster than the canonical PIMD. We used four path-integral beads, while the PI+GLE parameters were again taken from the GLE4MD web page, using *T* = 298 K and parameters: *N*_s_ = 6, *ℏ*ω_max_/*kT* = 50.

In total, 4000 ICs were selected for each type of sampling (Wigner, Wigner*, and QT). Electronic excitation energies for the three lowest singlet excited states and their oscillator strengths were calculated with XMS(4)-CASPT2(8/6)/def2-SVPD. A small fraction of ICs had to be discarded due to issues with electronic-structure convergence (see SI for details). Absolute photoabsorption cross-sections were calculated within the NEA as implemented in the Newton-X 2.0 package^61,62^ using a phenomenological Lorentzian broadening of 0.05 eV.

### 2.3 Excited-State Molecular Dynamics

TSH^15^ simulations were performed with SHARC 2.1,^54,55^ interfaced with BAGEL for the electronic-structure calculations. The TSH dynamics involved four singlet electronic states, and TSH trajectories were typically 25 fs long—the timescale was extended up to 100 fs for a small number of trajectories where the photolysis outcome was unclear within the first 25 fs. The time step for the nuclear dynamics was 0.5 fs, with 25 substeps for the propagation of the electronic quantities. The decoherence correction devised by Granucci and Persico^63^ was used to correct the TSH electronic populations. Nonadiabatic couplings were calculated with the wavefunction overlap scheme. After a successful hop, the kinetic energy was adjusted by rescaling the nuclear velocity vector isotropically. For each type of sampling (Wigner, Wigner*, and QT), the TSH dynamics was initiated from a subset of the 4000 ICs used for the NEA (see Table S1 for details about the numbers of TSH trajectories). We defined three narrow energy windows within each photoabsorption cross-section, centered around 5.00 eV (248 nm), 5.71 eV (217 nm), and 6.42 eV (193 nm). Each window had a total width of 0.3 eV. ICs were selected within a window if their transition energies fell within the energy range of the window. The *f*-biased selection scheme employed for some TSH simulations was applied by modifying the excite.py script in SHARC 2.1 (see details in Section 3). We implemented an *f*-biased selection with excitation probabilities proportional solely to the oscillator strengths. To calculate translational kinetic energy maps, the nuclear velocities of OH and CH_3_O fragments were collected after 25 fs of dynamics. No special treatment against ZPE leakage was applied given the very short timescale of the TSH simulations reported in this work (for a detailed discussion of the effect of ZPE in nonadiabatic dynamics, the interested reader is referred to ref (64)). The reader is referred to the SI for a comment about the discarded trajectories and their potential impact on the calculated quantum yields and kinetic energy maps.

## 3 Results and Discussion

### 3.1 Approximate Ground-State Nuclear Density of Methylhydroperoxide

**Figure 2:**
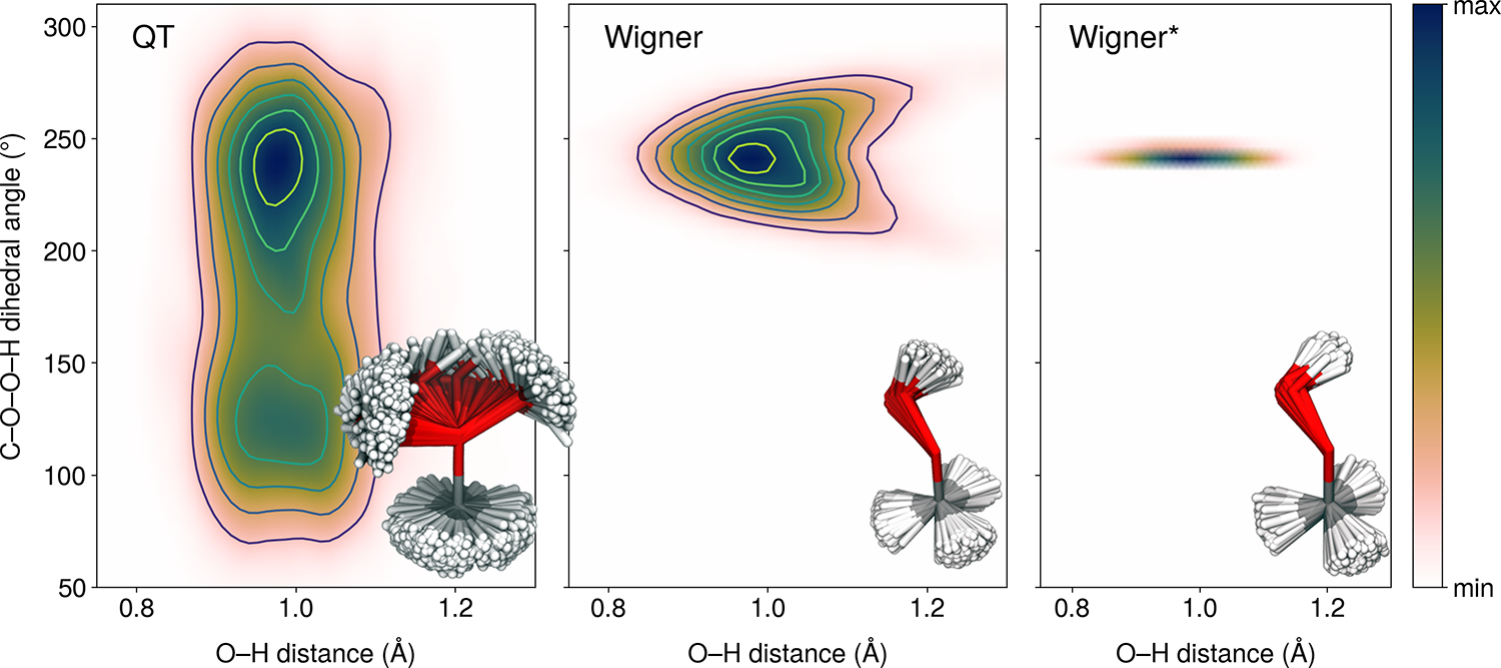
Distribution of the O–H distance and C–O–O–H dihedral angle of MHP for 4000 geometries sampled from: (left panel) ab initio molecular dynamics (MP2/aug-cc-pVDZ) using a quantum thermostat, (middle panel) Wigner distribution for uncoupled harmonic oscillators obtained from the MP2/aug-cc-pVDZ equilibrium geometry of MHP and corresponding harmonic frequencies, and (right panel) the same Wigner distribution but with the low-frequency C–O–O–H torsion removed from the sampling. The color maps were created by the Gaussian kernel density estimation using a 50 by 50 grid of points. The kernel bandwidth was estimated using Scott’s rule.^65^ The insets show the 4000 geometries sampled from each distribution, aligned with respect to the central C–O bond.

MHP is the simplest and most abundant organic peroxide in the atmosphere, with implications on atmospheric radical and oxidative balance.^45^ From a theoretical perspective, MHP exhibits an interesting low-frequency normal mode at 201 cm^–1^ (MP2/aug-cc-pVDZ) that critically affects the sampling of ICs.^44^ More specifically, the C–O–O–H torsional mode of MHP is poorly sampled when using a distribution built from linear normal modes—like the Wigner distributions constructed from the equilibrium geometry and vibrational modes obtained from quantum-chemical calculations—leading to an artificially broad distribution of O–H bond lengths. A significant number of MHP geometries sampled from a harmonic Wigner distribution (“Wigner”) exhibit O–H bond lengths larger than 1.2 Å (middle panel, Figure 2). This problem stems from the fact that atoms involved in low-frequency torsions are not per se rotated but moved along normal-mode vectors, which causes unphysical displacements of light H atoms. Torsions are inherently curvilinear and notoriously poorly represented by rectilinear normal modes with Cartesian displacements.^29,31,44^ The correlation between C–O–O–H torsion and O–H bond length is clearly visible in the middle panel of Figure 2, where torsion along the C–O–O–H mode around the equilibrium geometry is connected with an elongation of the O–H bond length. We note that sampling the same harmonic Wigner distribution at 300 K further enhances the artifact observed here at 0 K (see Figure S1 in the SI). Removing the C–O–O–H torsion from the Wigner sampling (“Wigner*”) immediately fixes the issue with the O–H bond length distribution (right panel of Figure 2—see also Figure S1 in the SI), but we will see later that this strategy is not without danger if the removed mode is of importance for the photochemistry/photophysics studied.

Sampling geometries from an ab initio molecular dynamics with QT leads to a proper distribution of the C–O–O–H dihedral angle and O–H bond length as these structural parameters are now coupled in the ab initio molecular dynamics (left panel of Figure 2). More importantly, QT reveals the much broader distribution of C–O–O–H dihedral angle, with the second energy minimum emerging at ∼120^°^. We note that this minimum in the ground electronic state of MHP is isoenergetic with that at ∼240^°^ and a much longer ab initio molecular dynamics with QT would be required to reach fully converged distributions. Structural differences between sampled ICs are obvious from the insets in Figure 2—QT samples almost free rotations around C–O and O–O bonds, while as mentioned above, these rotations are restricted in the Wigner sampling and even frozen in Wigner*. In principle, both minima of the ground-state potential energy surface could be sampled by calculating separate Wigner distributions. This extended sampling is, however, not necessary for the photochemical observables of interest here as the two minima correspond to chemically identical molecules from a symmetry perspective—with the minimum-energy geometries being mirror images of each other.

To verify the accuracy of QT, we compared its distribution for the C–O–O–H torsion angle and O–H bond length with fully converged path-integral results obtained with the PI+GLE approach (see Figure S1 in the SI). The QT and PI+GLE are in very good agreement, even for the highly anharmonic C–O–O–H mode, validating the distributions obtained with QT. We note that we also compared the distributions of nuclear momenta between Wigner, Wigner*, and QT (Figure S2 in the SI), showing overall a good agreement between the methods.

The incorrect description of the O–H bond length in the Wigner sampling will directly affect the calculated photoabsorption cross-section, σ(λ), for MHP. The sensitivity of σ(λ) on the accuracy of bond-length distributions lies in the fact that the low-lying singlet excited states of MHP exhibit an antibonding *n*σ* character (akin to other alkyl-peroxides^13^). The excitation energy of an electronic state exhibiting a *n*σ* character is generally highly sensitive to the length of the chemical bond(s) where the antibonding σ* orbital is localized. At the optimized ground-state geometry of MHP, the first excited electronic state (S_1_) has a *n′*σ*(O–O) character (see Figure 1 for a depiction of the molecular orbitals). It is followed, approximately 1 eV higher in energy, by two electronic states having a *n*σ*(O–O) and *n′*σ*(O–H) (see Table S3 in the SI). Hence, using a proper approximate ground-state nuclear density distribution is critical to ensure an accurate description of the *n′*σ*(O–H) transition for the calculated photoabsorption cross-section σ(λ) and also potentially for other observables as we will see below.

### 3.2 Photoabsorption Cross-Section of Methylhydroperoxide

**Figure 3:**
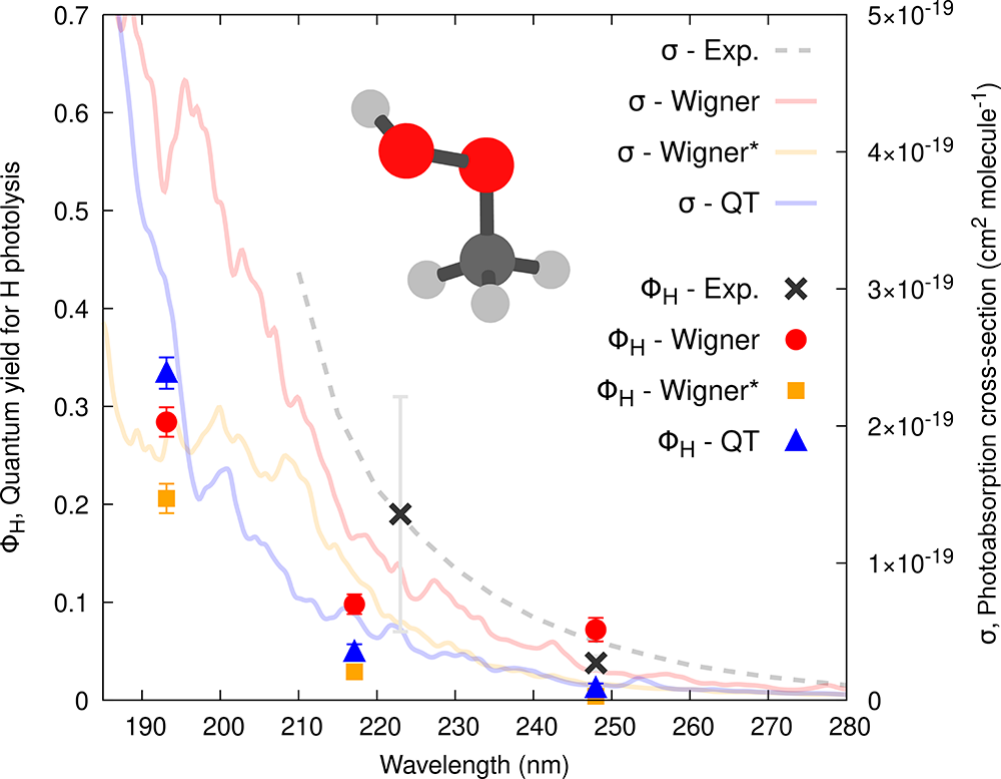
Calculated photoabsorption cross-sections (σ, curves) and wavelength-dependent quantum yields (ϕ_*H*_, symbols) for the H-atom photolysis from MHP. σ and ϕ_*H*_ were obtained from the NEA and TSH dynamics, respectively, based on a uniform selection of ICs generated from a Wigner distribution (red curve and circles), a Wigner distribution with the lowest-frequency mode removed (Wigner*, orange curve and squares), and a QT-based ab initio molecular dynamics (blue curve and triangles). The dashed curve and black crosses correspond to experimental data (see main text). Error bars represent the standard deviation for the calculated quantum yields or the reported error bars for the experimental values (obtained from refs (69, 70))—we note that the quantum yield value at 223 nm is deduced from the quantum yield of the CH_3_O radical (see main text for discussion). The insets show the molecular structure of MHP.

Our investigation of the role of IC sampling for photochemical observables begins with the photoabsorption cross-section of MHP, σ(λ). We focus more specifically on the low-energy tail of this quantity as this spectral region plays an important role in the context of atmospheric chemistry due to its overlap with the solar actinic flux. Figure 3 (right axis) compares the predicted σ(λ) (colored curves) using the three different sampling procedures for the NEA – Wigner (red), Wigner* (orange), and QT (blue) – to the experimental cross-section (grey dashed curve) obtained by combining data from refs (66, 67) as recommended in the MPI-Mainz UV/Vis Spectral Atlas.^68^ Note that the experimental photoabsorption cross-section appears smooth and structureless because of the dissociative (i.e., unbound) nature of the potential energy surfaces of the excited electronic states.

The fact that σ(λ) obtained with a Wigner sampling is closer to the experimental reference than that obtained with QT or Wigner* – both exhibiting a too low overall cross-section – may appear deceptive at first glance. However, the electronic structure method used in this work, namely XMS-CASPT2, exhibits too small oscillator strengths with respect to a reference method like CC3 or even LR-TDDFT (see SI for a detailed benchmark). This observation explains why combining QT sampling with LR-TDDFT for the transition energies and oscillator strengths provided a photoabsorption cross-section in excellent agreement with the experimental one in an earlier work.^44^ The increase of absorption intensities observed for the Wigner-based cross-section (low-energy tail) is an artifact caused by the broad O–H bond length distribution provided by this sampling method (see Figure 2) that causes the transitions of *n′*σ*(O–H) character (transitions with large oscillator strength) to fall down in energy. These intense transitions pollute the low-energy tail of the spectrum and increase the overall photoabsorption cross-section in this region (as discussed in detail in ref (44)). While we regard this effect as artificial, its impact on quantum yields is yet to be determined. Since the nonphysical stretching of the O–H bond is absent in QT and Wigner* sampling, their cross-sections in the tail region are smaller and smoother, being mainly built from *n*σ*(O–O) transitions of very weak intensity. Employing a method like CC3 to calculate the transition energies and oscillator strengths on the support of the QT- or Wigner*-sampled geometries would lead to a calculated photoabsorption cross-section in better agreement with the experimental one in terms of its intensity for the good reason and not due to an artifact as observed here with the Wigner-sampled geometries.

Focusing now on the higher-energy spectral range (we stress here that our calculations only include transitions toward the three lowest singlet excited electronic states), the differences observed between the results obtained with QT and Wigner* indicate that filtering out the problematic low-energy frequency from a standard Wigner sampling does not provide results equivalent to QT. Wigner* exhibits a broad (low-intensity) band at around 200 nm that does not appear in the QT spectrum. Extending the range of the photoabsorption cross-sections to 150 nm reveals that a broad high-intensity band at 170 nm in the Wigner and QT cross-sections appears much narrower when the Wigner* sampling is used (see Figure S3 in the SI). A scan of the potential energy curves along the C–O–O–H dihedral angle (see Figure S4 in the SI) reveals that the excited electronic states and their transition dipole moments with S_0_ are significantly affected by this torsion, conversely to the ground state. Hence, simply removing the torsion along the C–O–O–H dihedral angle from the sampling process, as done with Wigner*, may solve one issue (the artificial O–H bond lengths) but lead to an improper account of the role of the torsion in the photoabsorption of MHP. In other words, correcting a Wigner sampling by removing low-frequency torsions may be hazardous when these torsions potentially act as photoactive modes.

### 3.3 Wavelength-Dependent Quantum Yields of Methylhydroperoxide

Let us now concentrate on the influence of the ICs on the determination of wavelength-dependent quantum yields, ϕ(λ), calculated from the TSH simulations. We start by comparing ϕ(λ) obtained from a uniform selection of ICs sampled from the different distributions—Wigner, Wigner*, or QT. In the uniform selection, we selected ICs randomly from the specified distribution within each energy window, without applying any other filters. We will discuss later the results obtained from an *f*-biased sampling of the ICs, where the probability of selecting a particular IC is influenced by its oscillator strengths (see, for example, ref (62)).

MHP has two main photolysis channels—the photo-triggered release of a OH radical or an H atom. The other minor channels at higher excitation energies involve the photodissociation of an O atom (combined with the formation of methanol) or the simultaneous photodissociation of a H and O atom. The photolysis channel followed by the excited MHP molecule is mainly determined by the initial character of the excited electronic state reached by the light-absorption process. For the excitation wavelengths explored in this work, ϕ_*OH*_ + ϕ_*H*_ ≈ 1 such that we focus on the ϕ_*H*_ for our analysis (ϕ_*H*_ values being more directly related to the issues with ground-state distributions).

The experimental photolysis quantum yields for MHP were measured by Vaghjiani and Ravishankara at an excitation wavelength of 248 nm, corresponding to the edge of the low-energy tail of σ(λ) (see Figure 3).^69^ At this wavelength, ϕ_*H*_ = 0.038 ± 0.007 and ϕ_*OH*_ was estimated to be 1.00 ± 0.18. Thelen et al. measured the photodissociation at 193 and 248 nm using photofragment translational spectroscopy and did not observe H dissociation (this work does not report quantum yields).^71^ Blitz et al.^70^ measured a quantum yield for the CH_3_O radical of 0.81 ± 0.12 at 223 nm, which indirectly informs on the value of ϕ_*OH*_ (considering that only OH is formed from the photolysis of MHP at this wavelength). We connect this value to ϕ_*H*_ by 1 −ϕ_*OH*_, but note that this value should be taken cautiously.

We investigated the wavelength dependence of ϕ_*H*_ by defining three equidistant narrow energy windows centered at 248, 217, and 193 nm. These windows were used to select ICs from the three different ground-state sampling strategies. TSH simulations were then conducted based on these ICs, leading to the prediction of ϕ_*H*_ for each window and each sampling technique (Figure 3, left axis). The choice of ICs was labeled as uniform, meaning that all ICs with vertical transitions falling within the narrow excitation windows were accepted—the ensuing trajectories were calculated and treated as equally important events. In this way, ϕ_*H*_ was computed as *N*_H_^window^ / *N*^window^, where *N*_H_^window^ is the total number of trajectories starting from a given excitation window and following the H photodissociation pathway, while *N*^window^ is the total number of trajectories launched from a window, regardless of their outcome. The error bars were estimated following ref (28). The TSH results obtained from a Wigner sampling (TSH/Wigner in the following) differ from that obtained with Wigner* (TSH/Wigner*) and QT (TSH/QT) at all wavelengths. Not unexpectedly, the TSH/Wigner predicts a larger quantum yield for the H dissociation in the low-energy window. This behavior is directly correlated with the artificially broad O–H bond distribution created by a Wigner sampling and discussed in Section 3.1 and above for the case of photoabsorption cross-sections. TSH/Wigner* predicts almost no H dissociation (2 trajectories out of 487), while the TSH/QT quantum yield for this channel is slightly larger (7 trajectories out of 575). Hence, the ϕ_*H*_ values predicted by TSH/QT and TSH/Wigner* are somewhat smaller than the experimental reference (including its error bar), while ϕ_*H*_ predicted with TSH/Wigner is larger.

Considering that there are electronic transitions with high and low oscillator strengths within a given excitation window, one may wonder whether it is reasonable to assign them the same weight in the process of selecting ICs. Since the oscillator strength correlates with the light-absorption probability, one can test whether the nuclear configurations that have larger oscillator strengths should be preferably selected instead of those with low oscillator strengths (within a certain window). Such an *f*-biased selection was proposed in the literature^62^ and implemented in TSH codes such as Newton-X^61,62^ and SHARC.^54,55^ The *f*-biased selection of ICs implies that a given electronic transition, labeled *i*, is associated with a probability calculated as *f*_*i*_/*f*_max_, with *f*_*i*_ being the oscillator strength of this transition (alternatively, one can use the Einstein coefficient B, which is proportional to the square of the transition dipole moment). *f*_max_ corresponds to the most intense transition within the selection window. The probability is then compared to a randomly generated number in the [0,1] interval, and an IC is selected if its probability is larger than the random number. In this work, we used an *f*-biased selection based on oscillator strengths in a modified version of the SHARC code, and we note that a selection based on squared transition dipole moments was also proposed by Persico and Granucci.^28^ Since the oscillator strength differs from the squared transition dipole moment only by a factor proportional to the excitation energy, the two selection schemes are expected to differ only moderately when the sampling is performed within narrow energy windows.

**Figure 4:**
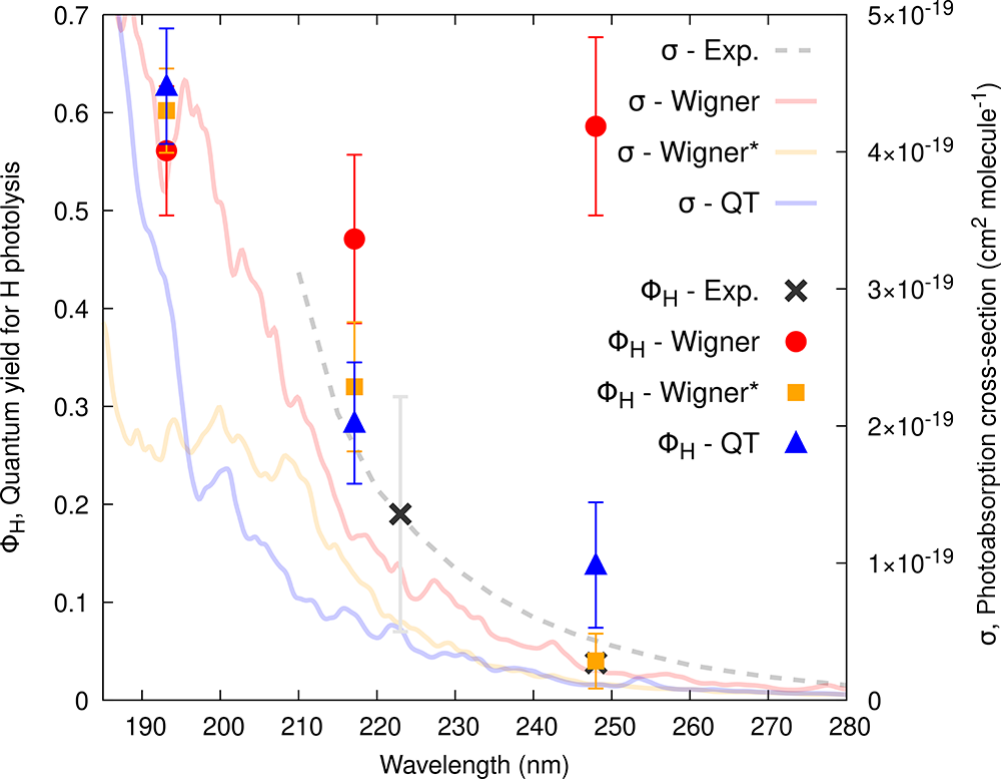
Calculated MHP photoabsorption cross-sections (σ) and H dissociation quantum yields (ϕ_*H*_) obtained from the NEA and TSH dynamics, respectively, based on an *f*-biased selection of ICs (see main text) and compared to experimental data. The same definitions as in Figure 3 apply for the colors, curves, and symbols.

The predicted ϕ_*H*_ obtained from TSH simulations initiated with an *f*-biased selection of the ICs are presented in Figure 4 (left axis). The error bars are significantly larger despite using the same pool of ICs as for the data in Figure 3. The *f*-biased selection would require to calculate a much larger pool of ICs with corresponding excitation energies and oscillator strengths to match the error bars of the uniform selection. The rejection of ICs is particularly strong when the energy window contains few very intense transitions and a large number of weak transitions. This behavior can potentially be problematic when transitions with an artificially high oscillator strength appear within the selection window, as we will see. The ϕ_*H*_ values calculated with the *f*-biased selection are very different from those based on the uniform strategy (compare Figure 4 with Figure 3). As expected from its definition, the *f*-biased selection amplifies the difference between TSH/Wigner, TSH/Wigner*, and TSH/QT for MHP, reflecting the issue caused by the artificially low-lying *n′*σ*(O–H) transitions. As a result, the ϕ_*H*_ values predicted by TSH/Wigner lie around 0.5 across the whole wavelength range. This observation raises a red flag for using an *f*-biased selection strategy when the ground-state sampling affects the balance between bright and dark transitions within a selection window. We note that the *f*-biased selection is often set as a default sampling strategy in many standard TSH codes. However, the problem does not lie with the *f*-biased selection per se, but its combination with an improper sampling technique that amplifies the errors. TSH/Wigner* and TSH/QT predict ϕ_*H*_ values that are more consistent with available experimental data although not in perfect agreement. The ϕ_*H*_ value obtained with TSH/QT at 248 nm is overestimated as rare transitions involving brighter *n′*σ*(O–H) are more likely to be selected than the majority of dark *n*σ*(O–O) present in this window. The TSH/Wigner* dynamics leads to ϕ_*H*_ values that appear closer to the available experimental values. Nevertheless, it is difficult to fully assess the *f*-biased selection algorithm without good-quality experimental data over the whole wavelength range. As noted above, we also need to keep in mind that the electronic-structure method used in this work, XMS-CASPT2, underestimates oscillator strengths, in particular for the lowest *n′*σ*(O–O) state.

An alternative to the *f*-biased selection strategy would consist in assigning a weight to each TSH trajectory initiated from a uniform selection of ICs. Effectively, this protocol means that a large number of TSH trajectories should be simulated, and the contribution of each TSH trajectory to the calculation of ϕ would be weighted by a factor determined from its IC. An earlier work^9^ proposed that ϕ(λ) can be calculated as σ_product_(λ)/σ_*tot*_(λ), where σ_product_(λ) is a photoabsorption cross-section obtained uniquely from the ICs that lead to a certain photoproduct, whereas σ_*tot*_(λ) is the total cross-section accounting for all ICs. Using a ratio of photoabsorption cross-sections is justified by the fact that σ(λ) is proportional to the number of photons absorbed at a wavelength λ, whereas the number of absorbed photons is proportional to the number of product molecules formed. If we focus on a narrow excitation window and ignore the broadening effects in the NEA expression for σ (see eq 2 in ref (44)), the estimate of ϕ reduces to ∑_*i*_*f*_*i*, product_^window^/∑_*i*_*f*_*i*_^window^, where ∑_*i*_*f*_*i*, product_^window^ is a sum over the oscillator strength of the ICs *i* within a window that lead to the formation of a certain product, and ∑_*i*_*f*_*i*_^window^ is the sum over the oscillator strength of all the ICs within this energy window. In other words, instead of counting the trajectories yielding a certain photoproduct as done earlier, we may sum up the oscillator strengths of their ICs and divide this sum by the total sum of oscillator strengths within the energy window under consideration. Using this strategy with our NEA and TSH data leads to values for ϕ_*H*_ that are similar to the values obtained from the *f*-biased selection (see Table S2 in the SI) (ultimately, these two schemes should not lead to completely identical results—in the *f*-biased selection strategy, the *f*_*i*_ are compared to *f*_*max*_ within a given energy window, while in the new scheme discussed here, the *f*_*i*_ are compared to the average *f* for the energy window of interest). This method, however, suffers from an issue in the low-energy tail of the photoabsorption cross-section, where a very small number of trajectories with large initial oscillator strengths (e.g., only 2/487 trajectories for Wigner* at 248 nm) results in relatively high ϕ_H_ values (e.g., 0.142 for Wigner* at 248 nm). The uncertainty for these values is very high and heavily depends on the accuracy of the oscillator strengths employed (a bottleneck for the electronic-structure method used in this work as discussed in the SI).

### 3.4 Translational Kinetic Energy Distribution for the OH Photolysis of Methylhydroperoxide

**Figure 5:**
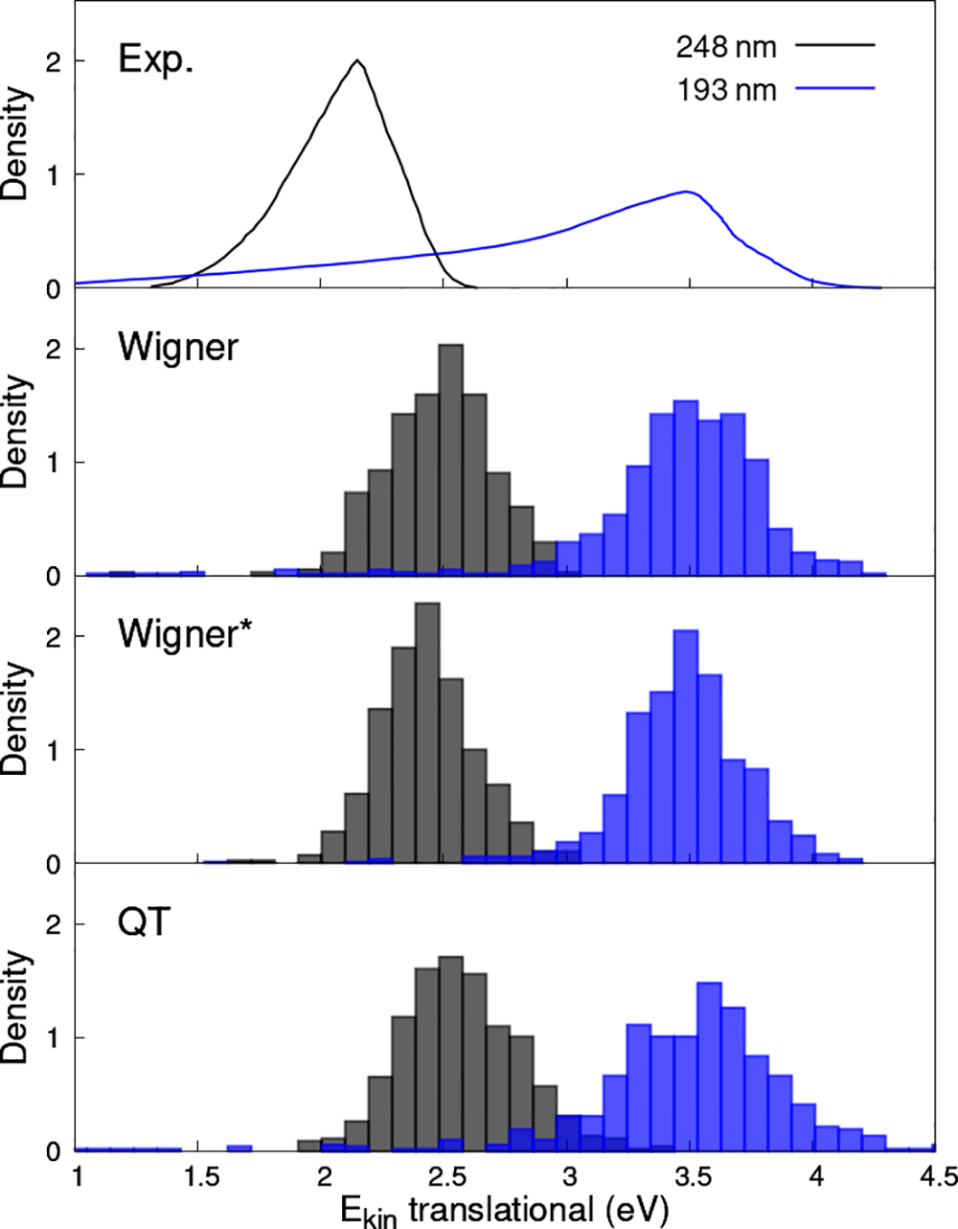
Translational kinetic energy maps for OH photodissociation of MHP. Experimental data sets for an excitation at 248 nm (black) and 193 nm (blue) (ref (71)) are compared to the theoretical results obtained from TSH simulations initiated from a Wigner, Wigner*, or QT sampling (with a uniform selection of the ICs).

The final observables considered in this work are translational kinetic energy distributions. Experimentally-derived data for MHP based on measurements in a cold molecular beam are available for OH dissociation at 193 and 248 nm^71^ and reproduced in the top panel of Figure 5. The large swarm of TSH trajectories that we generated to calculate ϕ(λ) allows us to estimate the translational velocities and the kinetic energies of the released OH and CH_3_O fragments. The results from TSH/Wigner, TSH/Wigner*, and TSH/QT samplings with the uniform selection of ICs are shown in the lower panels of Figure 5. Overall, the three types of sampling lead to very similar translational kinetic energy maps (Figure 5). In all cases, the density peaks are shifted toward higher energies with respect to those observed in the experimental maps. Such a shift can be partly explained by the electronic-structure method employed. XMS-CASPT2/def2-SVPD underestimates the OH ground-state dissociation limit by 0.17 eV when compared to UCCSD(F12*)(T)/aug-cc-pVQZ, which is consistent with the higher kinetic energies of the fragment following the photodissociation event (the ground and excited states are near degenerate in the dissociation limit). Also, our TSH trajectories are relatively short and the estimated kinetic energies may not be fully converged for all trajectories, i.e., fragments may still feel a weak interaction at the end of the simulation when the kinetic energy is determined.

An exciting and counter-intuitive feature of both theoretical and experimental distributions is the long tail at low kinetic energy that appears only for the 193 nm excitation energy. The experimental study of Thelen et al.^71^ lacks any explanation of this feature, while the theoretical work of Mahata and Maiti^72^ did not predict the tail. We analyzed the TSH trajectories leading to very low translational kinetic energies for the released OH and noticed that these OH fragments exhibit large vibrational amplitudes. The most common scenario observed for the creation of these vibrationally-excited OH fragments is an initial H photodissociation, followed by the O–O bond cleavage that happens due to a nonadiabatic interaction between the *n*σ*(O–H) and *n*σ*(O–O) states. Collision of the departing O and H atoms then creates the OH fragment with a high vibrational and low translational energy. Figure S5 provides an example of such a TSH trajectory. As these events usually start with a H cleavage, the corresponding initial excitation that could lead to such processes should possess a relatively large oscillator strength. Hence, we also determined translational kinetic energy maps using a weighted IC selection, which accounts for the initial oscillator strengths of the ICs. Since the *f*-biasing scheme used above did not lead to a sufficient number of OH trajectories for a meaningful analysis, we employ an a posteriori correction by assigning weights to the TSH trajectories obtained from the uniform selection. The translational kinetic energy maps calculated from this biased weighing are shown in the SI, Figure S6. The differences between the maps obtained with TSH/Wigner, TSH/Wigner*, and TSH/QT samplings become more pronounced, with the TSH/QT results appearing to be the closest to the experimental data. In all cases, the weighted theoretical maps show a further enhanced tail for the excitation at 193 nm leading to a closer agreement with experimental evidence although a full convergence of these results would require a significantly larger number of TSH trajectories. In any case, the results obtained for translational kinetic energy maps appear to advocate further a potential bias of the observables calculated from TSH simulations.

## 4 Conclusions

In summary, this work explored how ICs and their sampling influence the calculation of photochemical observables when using the NEA and TSH simulations. As the photochemical quantities determined in this work—namely photoabsorption cross-sections, wavelength-dependent quantum yields, and translational kinetic energy maps—are of potential use in atmospheric photochemistry, we use as a test case the photodynamics of the MHP molecule, which exhibits a complex electronic structure and challenges standard protocols used in computational photochemistry. The predicted observables appear to depend significantly on the choice of ICs, in particular when the approximation underlying a sampling strategy leads to artificial distortions of the molecule along photoactive modes. The impact of the ICs on the results of excited-state dynamics simulations highlighted here is not limited to surface hopping simulations but would apply to other mixed quantum/classical^25,73,74^ or Gaussian-based methods.^75−78^ Despite the limited amount of experimental data available and the approximate electronic structure used in present calculations, the TSH dynamics based on a QT sampling appears to provide more reliable results than the dynamics initiated from Wigner sampling though only when oscillator strengths are properly taken into account – either by biasing the selection of ICs or equivalently weighing the results at the end of the simulation. The benefit of biasing the selection of ICs was spotlighted for calculating the wavelength-dependent quantum yield for H photodissociation and the translational energy maps for OH photodissociation. From a photochemical perspective, the TSH/XMS-CASPT2 simulations presented in this work indicate that the low-energy tail in the translational kinetic energy maps is caused by nonadiabatic processes leading to the formation of a highly vibrationally excited OH fragment. The removal of low-energy normal modes, here a torsion, from the construction of a Wigner distribution leads to improved results for the photoabsorption cross-section at low energy but hampers an adequate description of this quantity at higher energy as the torsion affects high-energy electronic states. Hence, this work advocates a careful evaluation of the approximations underlying a sampling strategy for ICs used in excited-state dynamics, in particular when the low-energy modes of a molecule affect the electronic states of interest to its photochemistry.

## Supporting Information Available

A detailed benchmark of electronic-structure methods for the low-lying excited states of MHP, a comment on the discarded trajectories from the TSH simulations, tables containing the total number of TSH trajectories simulated for each sampling strategy, the raw data for the quantum yields presented in the main text, and the supporting figures mentioned in the main text (PDF)
