# Supplementary material for: Deciphering the Influence of Ground-State Distributions on the Calculation of Photolysis Observables
Source: J Phys Chem A. 2023 Aug 9;127(35):7400–9. doi: 10.1021/acs.jpca.3c02333 (PMC10493954; doi:10.1021/acs.jpca.3c02333)
Supplement: Supplementary file 1 — jp3c02333_si_001.pdf [file jp3c02333_si_001.pdf]

Supporting Information: Deciphering the Influence of Ground-State Distributions on the Calculation of Photolysis Observables

Antonio Prlj,*,†,‡ Daniel Hollas,*,† and Basile F. E. Curchod*,†

†*Centre for Computational Chemistry, School of Chemistry, University of Bristol, Bristol BS8 1TS, United Kingdom*

‡*Division of Physical Chemistry, Ruđer Bošković Institute, 10000 Zagreb, Croatia*

E-mail: antonio.prlj@irb.hr; daniel.hollas@bristol.ac.uk; basile.curchod@bristol.ac.uk

Contents

1 Additional computational details **S-2**

1.1 Electronic-structure benchmarks S-2

1.2 Discarded ICs and Trajectories S-3

2 Benchmarking electronic-structure methods for the description of MHP excited electronic states **S-7**

2.1 Reference CC3 results S-7

2.2 Performance of XMS-CASPT2 . . . . S-8

2.3 Accuracy of single-reference methods and LR-TDDFT S-9

3 Supplementary figures **S-10**

References **S-16**

1 Additional computational details

1.1 Electronic-structure benchmarks

We benchmarked the reliability of the XMS(4)-CASPT2(8/6)/def2-SVPD level of theory (see Table S3 below) by comparing the predicted vertical transition energies and oscillator strengths with those obtained with a larger aug-cc-pVTZ[S1] basis set, and against the reference CC3/aug-cc-pVTZ values. We have also tested other single-reference methods of various accuracy combined with basis sets of variable size. A discussion of the results can be found in Sec. 2 below.

Excited-state coupled cluster (CC) calculations[S2] were performed within the equation-of-motion (EOM) formalism in a hierarchy comprising CC2, EOM-CCSD, and CC3.[S3] All CC calculations were performed with the eT v1.8 program package.[S4]

Linear-response time-dependent density functional theory (LR-TDDFT)[S5,S6] was used

with the PBE0 exchange-correlation functional[S7] within the Tamm-Dancoff approximation (TDA).[S8] Calculations using ADC(2) (algebraic diagrammatic construction up to second order)[S9,S10] and its spin-component scaled variant (SCS-ADC(2))[S11] were performed with frozen core and the resolution of the identity approximation,[S12] using default SCS scaling factors (cos= 1.2, css= 0.33333). LR-TDDFT and ADC(2) excited-state calculations were done with Turbomole 7.4.1.[S13]

Turbomole was also employed for the ground-state estimate of the O – O dissociation limit. To determine this quantity, relaxed scan was performed with unrestricted Møller–Plesset perturbation theory up to second order (UMP2/aug-cc-pVDZ), while the energies were calculated using explicitly-correlated unrestricted coupled-cluster (CC) singles and doubles with perturbative triples, namely UCCSD(F12*)(T)/aug-cc-pVQZ.[S14]

1.2 Discarded ICs and Trajectories

A small fraction of ICs and TSH trajectories experienced numerical problems and their potential impact on calculated observables needs to be discussed.

Out of 4000 ICs from each type of sampling strategies, 81 ICs from Wigner sampling, 58 ICs from Wigner* sampling, and 136 ICs from QT sampling had problems with the convergence of oscillator strengths for either of the three excited states considered. These ICs were not used for the calculations of photoabsorption cross-sections or the TSH dynamics simulations. Considering that their number is relatively small and only an even smaller fraction of these ICs would have fit the narrow energy windows used for initiating TSH trajectories, we do not expect any large impact on calculated observables. We acknowledge that a small number of discarded ICs could potentially affect the fraction of H dissociation in the 248 nm window, since Φ_H(248 nm) depends on rare events. Nevertheless, after checking the discarded ICs that have transitions fitting in this window, we could not find any indication (based on excited-state characters and oscillator strengths) that they would lead to an excess H dissociation.

A fraction of TSH trajectories was discarded due to nonphysical discontinuities in total and/or electronic (potential) energies along the dynamics. Such issues originate from the multireference electronic structure method used (e.g., changes in active space along the trajectory). The number of discarded trajectories for each set of TSH simulations is given in Table S1. The number of discarded trajectories is negligible for the lowest energy window at 248 nm, while it amounts to around 10–15 % for the highest window at 193 nm. These trajectories were not used in the calculation of the quantum yields and velocity maps. In general, trajectories with total classical energy jumps larger than 15% of the current kinetic energy and nonphysical steps in electronic energies were discarded. However, if the discontinuities occurred after the outcome of photolysis was unambiguous (typically already after 10–15 fs of dynamics), trajectories were still accepted and used in the statistics of the quantum yields. Nevertheless, these trajectories were not used for the determination of the OH kinetic energy maps since OH velocities were collected at 25 fs. The number of OH trajectories with numerical problems occurring after the OH dissociation (but before 25 fs) was between 8 - 18% for all windows and all types of sampling, implying that their impact on kinetic energy maps should not be decisive in any case.

Table S1: Summary of the outcomes of the TSH simulations.

	**Wigner (uniform)**			**Wigner (f-biased)**		
window:	248 nm	217 nm	193 nm	248 nm	217 nm	193 nm
OH	440	815	675	12	18	24
H	34	88	259	17	16	32
O	-	-	1	-	-	1
O+H	-	1	9	-	-	-
discarded	4	21	109	1	2	4
total	478	925	1053	30	36	61
	**Wigner* (uniform)**			**Wigner* (f-biased)**		
window:	248 nm	217 nm	193 nm	248 nm	217 nm	193 nm
OH	485	890	560	48	34	51
H	2	26	140	2	16	75
O	-	-	-	-	-	-
O+H	-	1	5	-	-	2
discarded	-	18	112	-	1	14
total	487	935	817	50	51	142
	**QT (uniform)**			**QT (f-biased)**		
window:	248 nm	217 nm	193 nm	248 nm	217 nm	193 nm
OH	568	732	588	25	38	25
H	7	38	291	4	15	42
O	-	-	-	-	-	-
O+H	-	-	4	-	-	-
discarded	6	24	124	1	2	12
total	581	794	1007	30	55	79

Table S2: Photolysis quantum yields ϕ_H and the corresponding standard deviations as plotted in Fig. 3 and 4 in the main text. Note that ϕ_H values include a small fraction of H from the O+H dissociation channel.

	**Uniform selection**		
	248 nm	217 nm	193 nm
Wigner	0.072 ± 0.012	0.098 ± 0.010	0.284 ± 0.015
Wigner*	0.004 ± 0.003	0.029 ± 0.006	0.206 ± 0.015
QT	0.012 ± 0.005	0.049 ± 0.008	0.334 ± 0.016
	**f-biased selection**		
	248 nm	217 nm	193 nm
Wigner	0.586 ± 0.091	0.471 ± 0.086	0.561 ± 0.066
Wigner*	0.040 ± 0.028	0.320 ± 0.066	0.602 ± 0.043
QT	0.138 ± 0.064	0.283 ± 0.062	0.627 ± 0.059
	**A posteriori scaling**		
	248 nm	217 nm	193 nm
Wigner	0.631	0.506	0.564
Wigner*	0.142	0.312	0.603
QT	0.301	0.314	0.569

2 Benchmarking electronic-structure methods for the description of MHP excited electronic states

To assess the accuracy of the XMS(4)-CASPT2(8/6)/def2-SVPD level of theory that was used for our NEA and TSH simulations, we benchmarked the excitation energies and oscillator strengths at the Franck-Condon geometry, optimized using the MP2/aug-cc-pVDZ. The results are summarized in Table S3 and discussed below.

Table S3: Excitation energies and corresponding oscillator strengths (in parenthesis) for the first three excited singlet electronic states of MHP calculated with different electronic-structure methods. Energies are given in eV.

	$n'\sigma^*$(O–O)	$n\sigma^*$(O–O)	$n'\sigma^*$(O–H)
**EOM-CC3/aug-cc-pVTZ**	5.66 (0.00133)	6.93 (0.00461)	6.81 (0.01060)
EOM-CC3/cc-pVDZ	5.88 (0.00032)	7.17 (0.00218)	8.01 (0.01630)
EOM-CC3/aug-cc-pVDZ	5.64 (0.00161)	6.91 (0.00519)	6.71 (0.01063)
XMS-CASPT2(8/6)/def2-SVPD	5.72 (0.00019)	7.21 (0.00024)	7.23 (0.01046)
XMS-CASPT2(8/6)/aug-cc-pVTZ	5.52 (0.00035)	7.02 (0.00153)	6.84 (0.00978)
XMS-CASPT2(8/6)/def2-SVPD (real vertical shift set to 0.3 a.u.)	5.66 (0.00033)	7.11 (0.00108)	7.04 (0.01065)
EOM-CCSD/aug-cc-pVDZ	5.70 (0.00184)	7.02 (0.00612)	6.67 (0.01014)
EOM-CCSD/aug-cc-pVTZ	5.74 (0.00138)	7.05 (0.00554)	6.86 (0.01026)
EOM-CC2/aug-cc-pVDZ	5.64 (0.00439)	6.97 (0.01018)	6.13 (0.00800)
EOM-CC2/aug-cc-pVTZ	5.68 (0.00314)	7.01 (0.00933)	6.27 (0.00849)
ADC(2)/aug-cc-pVTZ	5.69 (0.00230)	7.01 (0.00828)	6.24 (0.00655)
SCS-ADC(2)/aug-cc-pVTZ	5.90 (0.00164)	7.21 (0.00728)	6.66 (0.00820)
LR-TDDFT/TDA/PBE0/aug-cc-pVDZ	5.47 (0.0009)	6.81 (0.0064)	6.46 (0.0123)

2.1 Reference CC3 results

The CC3 method with an aug-cc-pVTZ basis set was used as our reference, as CC3 was previously shown to produce highly accurate results for excitation energies and oscillator strengths for a large number of molecular systems.[S15] Comparing the CC3 results for aug-cc-pVDZ and aug-cc-pVTZ allows us to assess the convergence of the results with respect

to the basis set, with the largest difference for the electronic energy being 0.1 eV for the $n'\sigma^*$(O−H) state. The partial Rydberg character of this electronic state is revealed by comparing results obtained from a basis set without diffuse functions, cc-pVDZ, leading to a 1.3 eV shift in energy for this electronic transition. The partial Rydberg character of this electronic state is consistent with the conclusions from Ref. S16 concerning the electronic excitations of *tert*-butylhydroperoxide.

We note that there is also a pronounced basis set dependence of the oscillator strengths of the valence $n\sigma^*$(O−O) and $n'\sigma^*$(O−O) states. Increasing the basis set size from aug-cc-pVDZ to aug-cc-pVTZ decreases the oscillator strength by 25%. It is thus possible that there is a small residual error for oscillator strengths of these states even with the triple-zeta basis.

2.2 Performance of XMS-CASPT2

In our TSH simulations using XMS-CASPT2, we used a relatively modest def2-SVPD basis set. This choice has been dictated by the compromise between accuracy of the electronic-structure method and its computational cost. In terms of the excited-state energies, using an aug-cc-pVTZ basis set with XMS-CASPT2 yields transition energies that are closer to the CC3 reference than XMS-CASPT2/def2-SVPD. With def2-SVPD, the excitation energies are within 0.1 eV of the reference for the $n\sigma^*$(O−O) states, but overestimated by 0.4 eV for the $n'\sigma^*$(O−H) state. These results offer a validation for the XMS-CASPT2 strategy employed in our work, even if our results could have been improved by using a larger basis set – the cost of such calculations are currently too high to be combined with on-the-fly TSH dynamics.

A very important observation from our benchmark is that XMS-CASPT2 severely underestimates the oscillator strengths of the $n\sigma^*$(O−O) and $n'\sigma^*$(O−O) states, even with aug-cc-pVTZ. In principle, our TSH simulations should be insensitive to the absolute values of the oscillator strengths as long as the deviations are uniform for all electronic state con-

sidered. This is unfortunately not the case for MHP — oscillator strengths of the $n\sigma^*$(O–O) and $n'\sigma^*$(O–O) states are severely underestimated, even more so with the smaller def2-SVPD basis, while the (much larger) oscillator strength of $n'\sigma^*$(O–H) is very close to the reference. When it comes to the calculation of the photoabsorption cross-section for MHP, the severe underestimation of the oscillator strength for the $n\sigma^*$(O–O) and $n'\sigma^*$(O–O) transitions leads to a too low cross-section in the low-energy tail, as observed and discussed in the main text.

2.3 Accuracy of single-reference methods and LR-TDDFT

Among all the other electronic-structure methods tested, only EOM-CCSD was able to provide quantitatively consistent results for both energies and oscillator strengths across all excited states considered. Both CC2 and ADC2 severely underestimate the energy of the $n'\sigma^*$(O–H) transition. The inaccuracy of CC2 and ADC(2) for Rydberg states has been reported in the literature.[S17] This limitation can be alleviated by using the SCS scheme (as in SCS-ADC(2)), at the cost of deteriorating the description of the electronic energies for the pure valence states (by up to 0.2 eV). This observation is again consistent with previous findings, see for example Table 1 in Ref. S18.

Interestingly, CC2 (and to a lesser extent ADC(2)) overestimates the oscillator strengths of the $n\sigma^*$(O–O) and $n'\sigma^*$(O–O) transitions by a factor of two, even though the excitation energies of these states are close to the reference results. This observation again contrasts with the significant underestimation of these quantities by XMS-CASPT2.

We have also tested LR-TDDFT/TDA using the PBE0 functional. Similarly to CC2 and ADC2, the energy of the $n'\sigma^*$(O–H) transition with the partial Rydberg character is underestimated, while the energies of the other two electronic states are within 0.2 eV from the reference. Overall, the oscillator strengths obtained with LR-TDDFT/TDA/PBE0 show a more balanced agreement with the CC3 reference values than XMS-CASPT2.

3 Supplementary figures

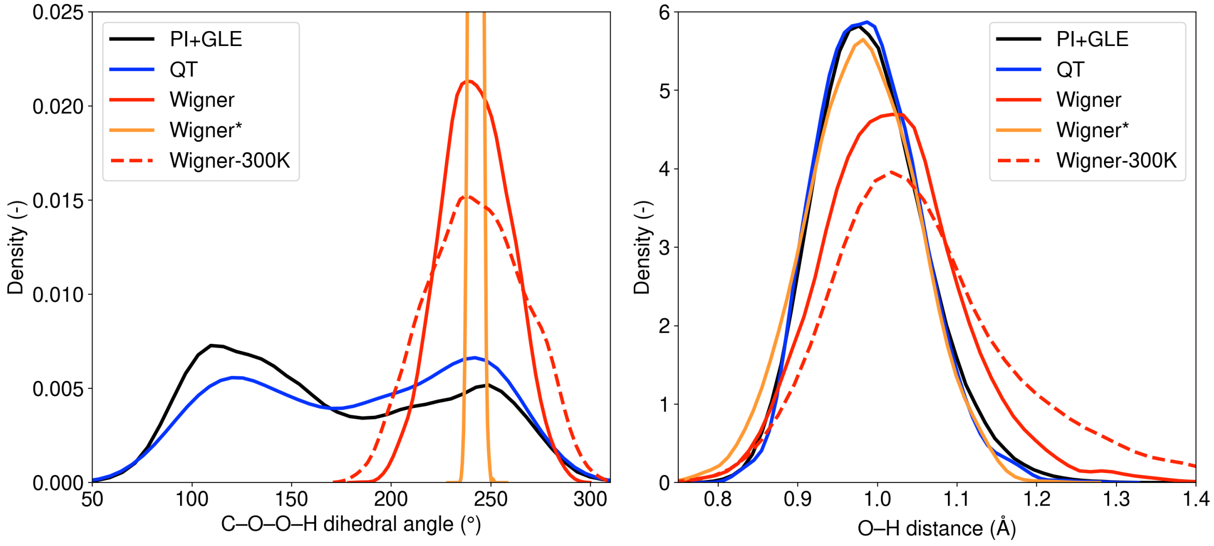

Figure S1: Ground-state density distribution for the C−O−O−H dihedral angle (left panel) and O−H distance (right panel) of MHP obtained from different sampling procedures, namely Wigner, Wigner*, QT, PI+GLE, and Wigner at 300 K (Wigner-300K). The data presented for the QT, Wigner, and Wigner* distributions correspond to the data shown in Fig. 2 from the main text. The distributions were smoothed using a Gaussian kernel density estimation and the kernel bandwidth was estimated using Scott's rule.[S19]

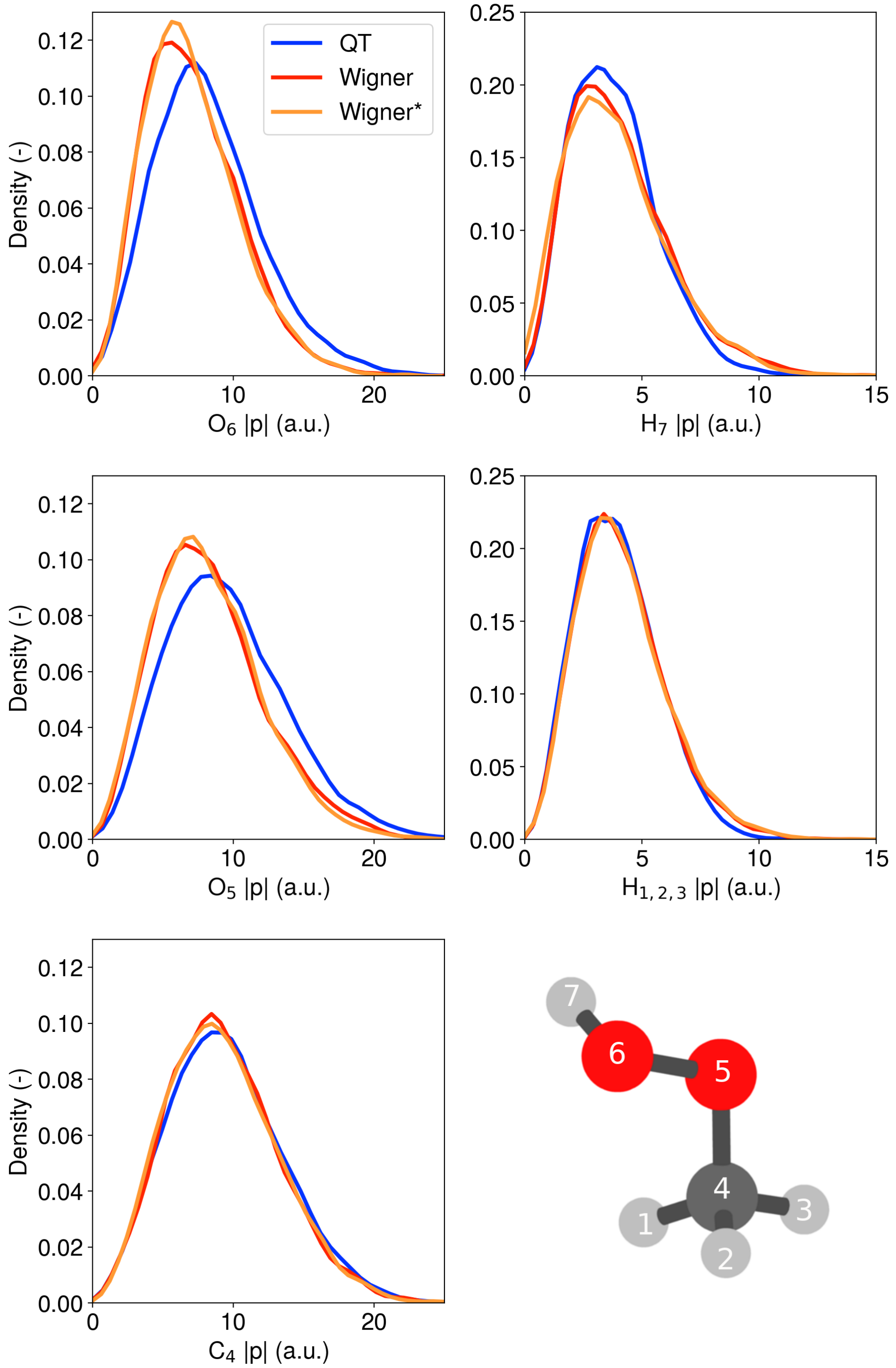

Figure S2: Nuclear momentum distributions obtained from different approximate ground-state sampling strategies. The plots were obtained by analyzing the nuclear momenta for the different sets of 4000 ICs discussed in the main text. The distributions were smoothed using a Gaussian kernel density estimation and the kernel bandwidth was estimated using Scott's rule.[S19]

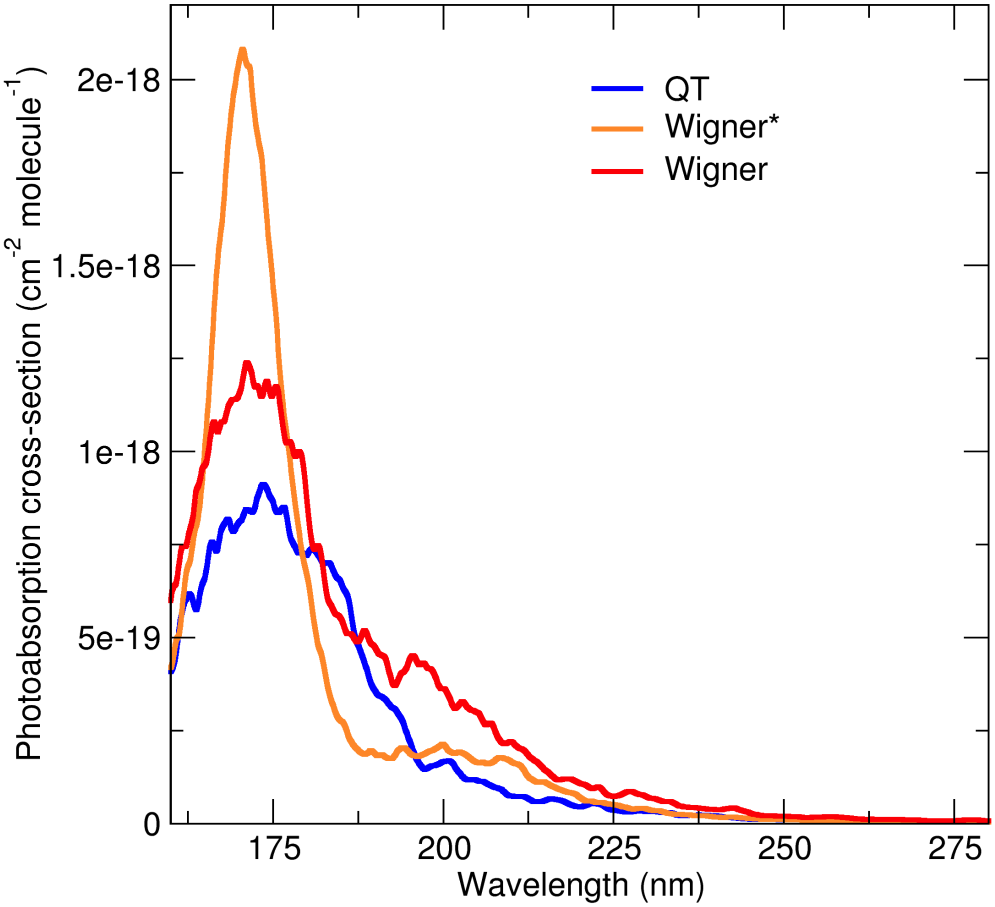

Figure S3: Calculated photoabsorption cross-sections for MHP. These cross-sections are the same as those presented in the main text, but the range at short wavelength was extended to better visualize the narrower band centered at 170 nm obtained with geometries sampled from Wigner*.

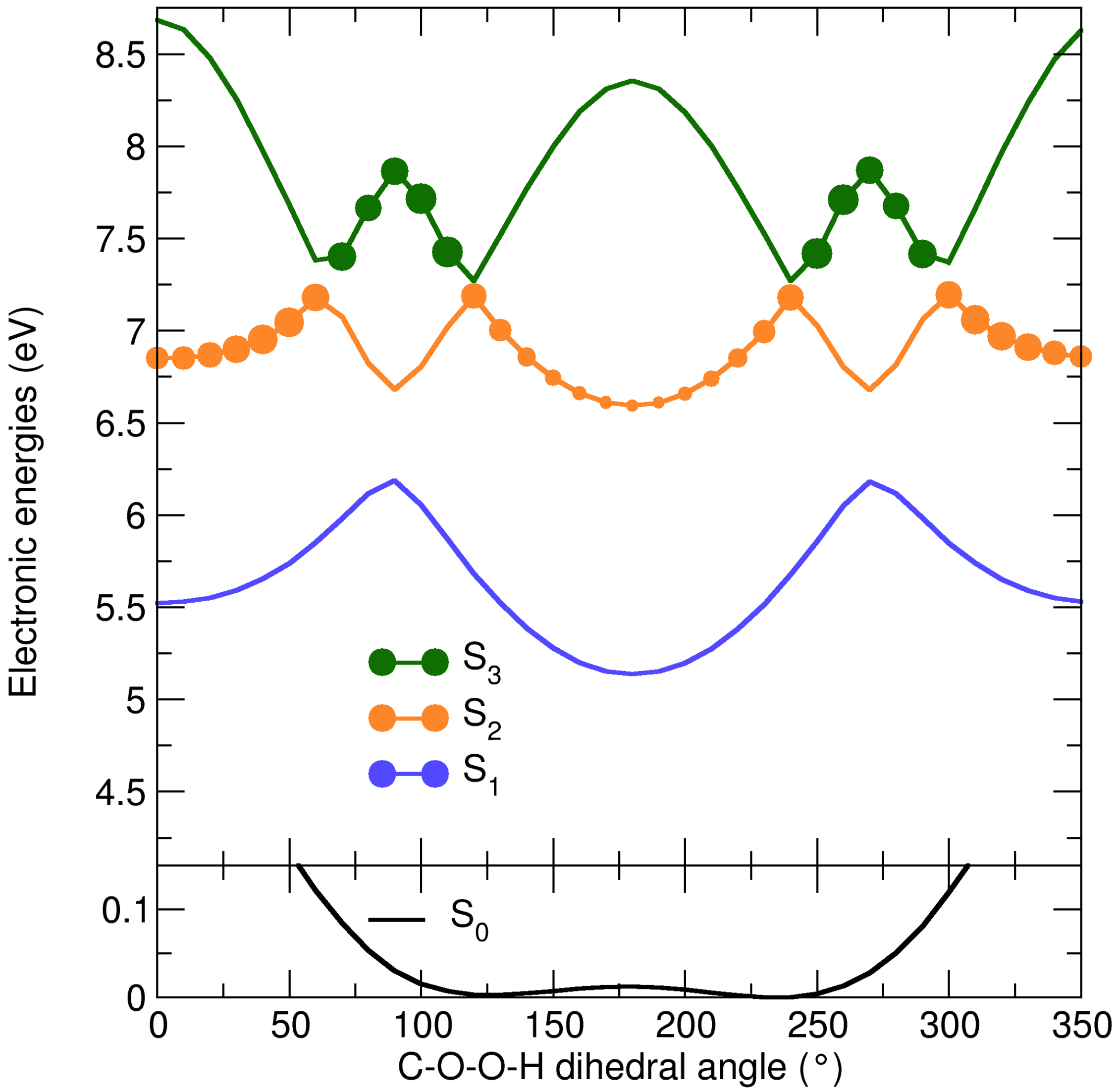

Figure S4: Rigid scan along the C−O−O−H dihedral angle of MHP. The reference geometry was obtained at the MP2/aug-cc-pVDZ level of theory. XMS-CASPT2(8/6)/def2-SVPD was used for the electronic energies and oscillator strengths. Filled circles are used to symbolize oscillator strength between S_0 and the (adiabatic) electronic state considered. The size of these circles is directly proportional to the size of the oscillator strength – when the oscillator strength is negligible (for example, $S_0 \rightarrow S_1$), only the curve for the electronic energy is visible. For sizeable oscillator strengths (for example, $S_0 \rightarrow S_2$ or $S_0 \rightarrow S_3$ depending on the region of the scan), the circles are clearly visible and highlight the exchange of electronic character between the adiabatic electronic states. More specifically, even a small variation of the C−O−O−H dihedral angle around one of the two S_0 minima leads to an important change in the electronic energy of the electronic-state character leading to a large oscillator strength. It is important to note that the normal mode corresponding to the C−O−O−H torsion is removed from the Wigner*, which explains the narrow high-intensity band in the Wigner* photoabsorption cross-section at around 170 nm ($\sim$ 7.3 eV) in comparison to the cross-sections obtained with the other sampling strategies (see Fig. S3).

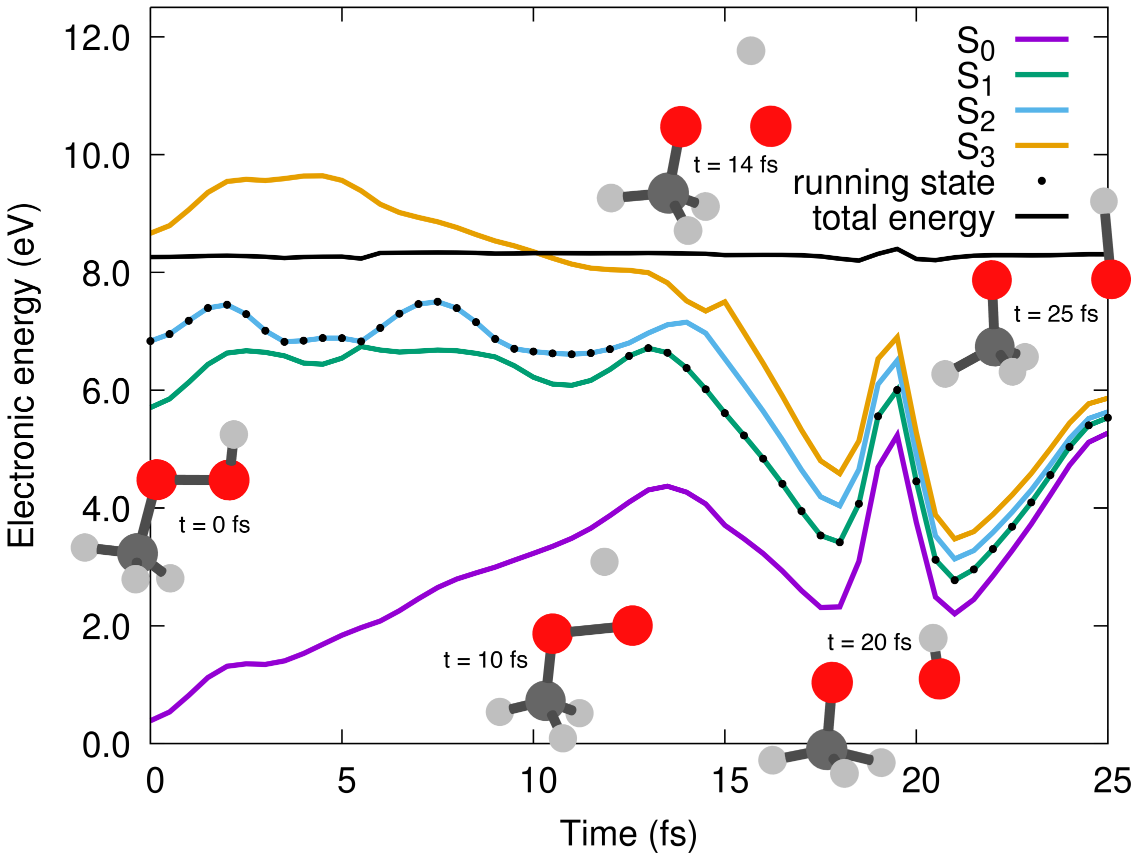

Figure S5: Electronic energies along an exemplary TSH trajectory depicting the nonadiabatic process responsible for the low kinetic-energy tail in the translational kinetic energy map for OH photodissociation in the excitation window around 193 nm. MHP is initially excited into S_2 of the $n'\sigma^*$(O−H) character and rapidly suffers an H photodissociation ($t = 10$ fs). Before the H dissociation is complete though, the molecule switches into S_1 of the $n'\sigma^*$(O−O)) character, which triggers the O−O bond cleavage ($t = 14$ fs). The O and H then recombine ($t = 20$ fs) to form a highly vibrationally excited OH fragment, as indicated by the strong variation in electronic energy when the OH reforms between 15–20 fs and the large amplitude oscillations of the O−H bond in the molecular snapshots at $t = 20$ fs and $t = 25$ fs.

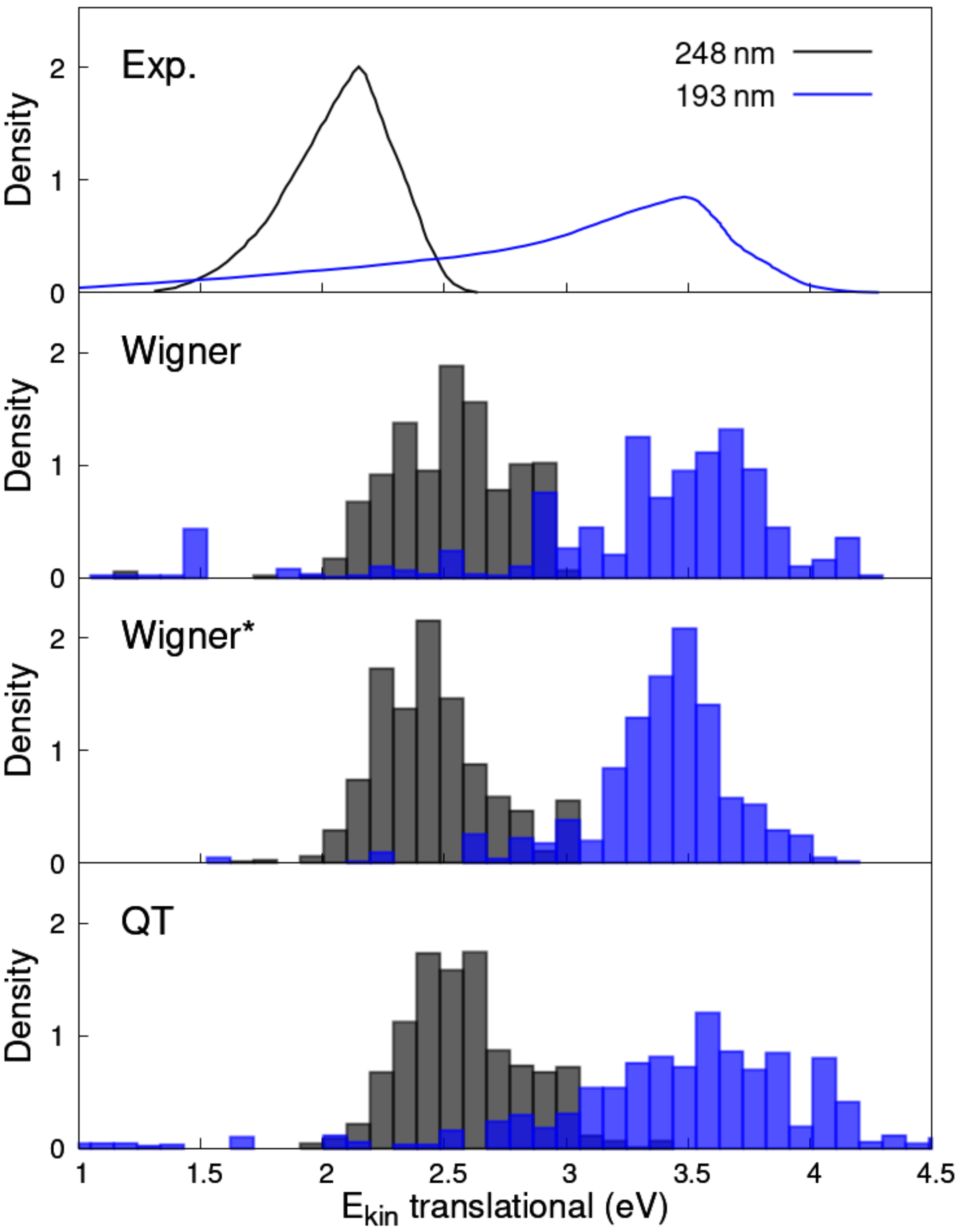

Figure S6: Translational kinetic energy maps for OH photodissociation. Experimental data sets for an excitation at 248 nm (black) and 193 nm (blue) (Ref. S20) are compared to the theoretical results obtained from TSH simulations initiated from a Wigner, Wigner* or QT sampling. The initial conditions were chosen using the uniform selection, as in Fig. 5 in the main text, but the final contribution of each TSH trajectory was weighted by the initial oscillator strength (see main text for details).

References

(S1) Woon, D. E.; Dunning Jr., T. H. Gaussian basis sets for use in correlated molecular calculations. III. The atoms aluminum through argon. *J. Chem. Phys.* **1993**, *98*, 1358–1371.

(S2) Koch, H.; Christiansen, O.; Jørgensen, P.; Olsen, J. Excitation energies of BH, CH2 and Ne in full configuration interaction and the hierarchy CCS, CC2, CCSD and CC3 of coupled cluster models. *Chem. Phys. Lett.* **1995**, *244*, 75–82.

(S3) Paul, A. C.; Myhre, R. H.; Koch, H. New and efficient implementation of CC3. *J. Chem. Theory Comput.* **2020**, *17*, 117–126.

(S4) Folkestad, S. D.; Kjønstad, E. F.; Myhre, R. H.; Andersen, J. H.; Balbi, A.; Coriani, S.; Giovannini, T.; Goletto, L.; Haugland, T. S.; Hutcheson, A.; Høyvik, I.-M.; Moitra, T.; Paul, A. C.; Scavino, M.; Skeidsvoll, A. S.; Tveten, Å. H.; Koch, H. eT 1.0: An open source electronic structure program with emphasis on coupled cluster and multilevel methods. *J. Chem. Phys.* **2020**, *152*, 184103.

(S5) Runge, E.; Gross, E. K. U. Density-Functional Theory for Time-Dependent Systems. *Phys. Rev. Lett.* **1984**, *52*, 997–1000.

(S6) Casida, M. E. *Recent Advances In Density Functional Methods, Part I*; World Scientific, 1995; pp 155–192.

(S7) Adamo, C.; Barone, V. Toward reliable density functional methods without adjustable parameters: The PBE0 model. *J. Chem. Phys.* **1999**, *110*, 6158–6170.

(S8) Hirata, S.; Head-Gordon, M. Time-dependent density functional theory within the Tamm–Dancoff approximation. *Chem. Phys. Lett.* **1999**, *314*, 291–299.

(S9) Trofimov, A. B.; Schirmer, J. An efficient polarization propagator approach to valence electron excitation spectra. *J. Phys. B* **1995**, *28*, 2299–2324.

(S10) Dreuw, A.; Wormit, M. The algebraic diagrammatic construction scheme for the polarization propagator for the calculation of excited states. *Wiley Interdiscip. Rev. Comput. Mol. Sci.* **2015**, *5*, 82–95.

(S11) Hellweg, A.; Grün, S. A.; Hättig, C. Benchmarking the performance of spin-component scaled CC2 in ground and electronically excited states. *Phys. Chem. Chem. Phys.* **2008**, *10*, 4119–4127.

(S12) Weigend, F.; Köhn, A.; Hättig, C. Efficient use of the correlation consistent basis sets in resolution of the identity MP2 calculations. *J. Chem. Phys.* **2002**, *116*, 3175–3183.

(S13) Furche, F.; Ahlrichs, R.; Hättig, C.; Klopper, W.; Sierka, M.; Weigend, F. Turbomole. *Wiley Interdiscip. Rev. Comput. Mol. Sci.* **2014**, *4*, 91–100.

(S14) Hättig, C.; Tew, D. P.; Köhn, A. Communications: Accurate and efficient approximations to explicitly correlated coupled-cluster singles and doubles, CCSD-F12. *J. Chem. Phys.* **2010**, *132*, 231102.

(S15) Loos, P.-F.; Scemama, A.; Blondel, A.; Garniron, Y.; Caffarel, M.; Jacquemin, D. A mountaineering strategy to excited states: Highly accurate reference energies and benchmarks. *J. Chem. Theory Comput.* **2018**, *14*, 4360–4379.

(S16) Prlj, A.; Ibele, L. M.; Marsili, E.; Curchod, B. F. E. On the theoretical determination of photolysis properties for atmospheric volatile organic compounds. *J. Phys. Chem. Lett.* **2020**, *11*, 5418–5425.

(S17) Kánnár, D.; Tajti, A.; Szalay, P. G. Accuracy of Coupled Cluster Excitation Energies in Diffuse Basis Sets. *J. Chem. Theory Comput.* **2017**, *13*, 202–209.

(S18) Tajti, A.; Tulipán, L.; Szalay, P. G. Accuracy of Spin-Component Scaled ADC(2) Excitation Energies and Potential Energy Surfaces. *J. Chem. Theory Comput.* **2020**, *16*, 468–474.

(S19) Scott, D. W. *Multivariate Density Estimation: Theory, Practice, and Visualization*; John Wiley & Sons, Inc., 1992.

(S20) Thelen, M.-A.; Felder, P.; Huber, J. R. The photofragmentation of methyl hydroperoxide CH_3OOH at 193 and 248 nm in a cold molecular beam. *Chem. Phys. Lett.* **1993**, *213*, 275–281.
